# Artificial Intelligence-Based Detection of Human Embryo Components for Assisted Reproduction by In Vitro Fertilization

**DOI:** 10.3390/s22197418

**Published:** 2022-09-29

**Authors:** Abeer Mushtaq, Maria Mumtaz, Ali Raza, Nema Salem, Muhammad Naveed Yasir

**Affiliations:** 1 Department of Primary and Secondary Healthcare, Lahore 54000, Pakistan; 2 Electrical and Computer Engineering Department, Effat College of Engineering, Effat University, Jeddah 22332, Saudi Arabia; 3 Department of Computer Science, University of Narowal, Narowal 51600, Pakistan

**Keywords:** in vitro fertilization (IVF), artificial intelligence (AI), deep learning, embryonic analysis, embryology, blastocyst imaging, embryo component segmentation network (ECS-Net)

## Abstract

Assisted reproductive technology is helping humans by addressing infertility using different medical procedures that help in a successful pregnancy. In vitro fertilization (IVF) is one of those assisted reproduction methods in which the sperm and eggs are combined outside the human body in a specialized environment and kept for growth. Assisted reproductive technology is helping humans by addressing infertility using different medical procedures that help in a successful pregnancy. The morphology of the embryological components is highly related to the success of the assisted reproduction procedure. In approximately 3–5 days, the embryo transforms into the blastocyst. To prevent the multiple-birth risk and to increase the chance of pregnancy the embryologist manually analyzes the blastocyst components and selects valuable embryos to transfer to the women’s uterus. The manual microscopic analysis of blastocyst components, such as trophectoderm, zona pellucida, blastocoel, and inner cell mass, is time-consuming and requires keen expertise to select a viable embryo. Artificial intelligence is easing medical procedures by the successful implementation of deep learning algorithms that mimic the medical doctor’s knowledge to provide a better diagnostic procedure that helps in reducing the diagnostic burden. The deep learning-based automatic detection of these blastocyst components can help to analyze the morphological properties to select viable embryos. This research presents a deep learning-based embryo component segmentation network (ECS-Net) that accurately detects trophectoderm, zona pellucida, blastocoel, and inner cell mass for embryological analysis. The proposed method (ECS-Net) is based on a shallow deep segmentation network that uses two separate streams produced by a base convolutional block and a depth-wise separable convolutional block. Both streams are densely concatenated in combination with two dense skip paths to produce powerful features before and after upsampling. The proposed ECS-Net is evaluated on a publicly available microscopic blastocyst image dataset, the experimental segmentation results confirm the efficacy of the proposed method. The proposed ECS-Net is providing a mean Jaccard Index (Mean JI) of 85.93% for embryological analysis.

## 1. Introduction

Infertility is a worldwide serious concern considering various points of view, and it needs attention. Infertility is alarmingly affecting almost 8–12% of couples worldwide [1]. Almost 80 million couples face infertility situations worldwide involuntarily. The prevalence of primary infertility ranges from 3 to 30%, and secondary infertility is almost double that of primary infertility [2]. According to the World Health Organization (WHO), the prevalence of infertility in India ranges from 3.9% to 16.8%. By the year 1981, almost 17.9 Million couples were facing infertility combining both primary and secondary infertility [3]. Similar to the worldwide prevalence, Canada’s infertility ranges from 12% to 16%, this cardiovascular risk factor is associated due to hypertension and it is affecting almost 5% of pregnancies [4]. Infertility causes frustration, anger, sadness, depression, and it is understood that infertility creates psychological distress in the partners with high social and emotional impacts [5].

The technique that involves the manipulation of the embryo outside the human body is known as assisted reproductive technology (ART). ART medical procedures are utilized to treat infertility issues, and it deals with both sperms and eggs. In vitro fertilization (IVF) is a well known, common ART in which the sperm and eggs are combined outside the human body and later fertilized ones are transferred back to the uterus [6]. IVF has now become an effective tool to address infertility with a contribution of almost 8 million successful pregnancies since 1978. Almost four decades of continuous research and finally concluded IVF is a safe ART for couples suffering from infertility. The embryo viability is a serious concern in which the morphological properties are checked and these viable embryos are used to transfer into the patient’s uterus [7].

**Figure 1:**
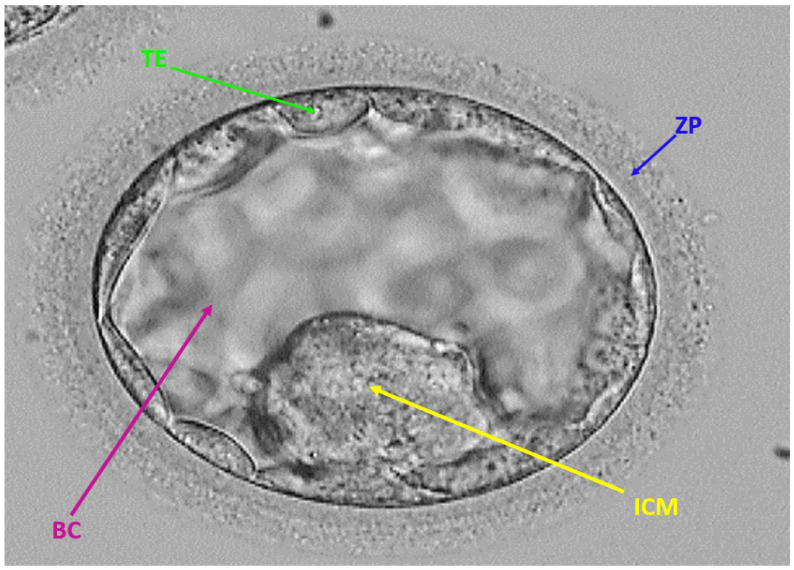
Embryonic components at the blastocyst stage. Inner cell mass (ICM), blastocoel (BC), trophectoderm (TE), and zona pellucida (ZP).

Previously in vitro fertilization was based on multiple embryo transfers to ensure the pregnancy. This multiple embryo transfer involves serious gestational issues for mothers and babies due to multiple pregnancies, therefore, one or two viable embryos are selected based on specific mythologies of the components [8]. The embryo viability test is an important process that involves manual analysis of embryo components and the embryos with specific morphological properties are selected for further process. To avoid the chance of multiple pregnancies the embryologist manually analyzes the embryo along with its components to pick the most viable ones, this process is time-consuming and requires expert-level knowledge with keen observation [9]. Almost on the fifth day the embryo forms into a blastocyst with the components of inner cell mass (ICM), blastocoel (BC), trophectoderm (TE), and zona pellucida (ZP). These components play an important role in a specific morphology that is analyzed by expert embryologists [10]. These embryo components are shown in Figure 1.

ICM is an embryo component that is formed inside the blastocyst before the transfer of the embryo to the uterus. It is located on one side of the embryo and it forms the trophoblast layer outside. The quality of ICM plays an important role in the success of in vitro fertilization [11,12]. BC is the cavity that is filled with fluid and it represents the formation of the blastocyst and the ICM is positioned in the corner of the blastocyst. BC creation shows the blastocyst creation on the fifth day, and its creation and specific morphology are important for successful IVF [13]. TE is the embryo component that creates the fluid to form the placenta. The TE later forms the ICM and the quality and formation are important to be analyzed before the embryo is transferred back to the uterus [14]. ZP is an important component that binds egg sperm and the thickness of the ZP is an important tool to check embryo quality. The thickness of the ZP starts to reduce as the embryo develops with time [15]. The manual observation of these components (ICM, BC, TE, and ZP) is time-consuming and requires efforts to detect the changes compared to the previous day.

Deep learning has the potential to help medical practitioners to achieve robust diagnostic solutions by effective learning from the training examples [16,17,18]. Deep-learning-based semantic segmentation of these embryo components can aid embryologists to analyze morphometric properties of these components for viable embryo selection. This study proposes embryo components segmentation network (ECS-Net) that performs deep-learning-based semantic segmentation to accurately detect these components for embryo analysis. The ECS-Net is based on two streams of base convolutional block and the separable convolutional block, these block extracts the important features that are later concatenated to produce a rich dense feature for better segmentation performance.

## 2. Background and Related Work

Semantic segmentation is considered an effective tool for pixel-wise classification of the image. Many computer vision applications are based on semantic segmentation including medical image segmentation [19,20,21]. In the case of embryology semantic segmentation can provide accurate pixel-wise detection of the embryo components. However, very few researchers focused on deep-learning-based embryological analysis. Considering non-segmentation-based methods for embryo analysis, Coticchio et al. used artificial intelligence to predict blastocyst creation. In detail, they utilized a K-Nearest neighbor, long short-term memory neural network and hybrid ensemble classifier to predict blastocyst creation through cytoplasm movement velocity recording [22]. Swada et al. proposed an artificially intelligent approach that predicts the probability of live birth using a specialized deep learning network [23].

Considering the embryo component detection for embryological analysis, these techniques provide more insight and deeper analysis opportunities as they provide the morphology of these components. Most embryo analysis schemes are based on single-component detection. A non-learning-based level-set segmentation approach is utilized by Singh et al. to segment the TE region from the image. They used preprocessing of the image using retinex filtering and used morphological operations to enhance the segmentation performance [24]. Kheradmand et al. used a 16-layered fully convolutional network to detect ICM, they used preprocessing steps to enhance the image before feeding it to the network [25]. Another deep learning approach is used to detect ICM effectively from the embryo image is proposed by Rad et al. First they utilized the famous U-Net approach, and they created the dilated version of the U-Net by using several dilated convolutions to increase the receptive field. The multi-resolution ensemble is created with the stacked dilated U-Net by using four different resolutions [26]. Similarly considering single component detection, the same group used an ensemble approach to detect ZP from the embryo microscopic images. They used a hierarchical scheme, in which a mixed scheme of pixel-level and global-level is used to detect the inner boundary of the ZP, then a patch-based approach is used to feed the hierarchical network with complementary learning for ZP refinement [27]. Rad et al. proposed an effective deep learning method to detect TE. They developed four different deep learning models for TE detection, and a multi-scale deep learning model is produced that combines all five models in a multi-scale scenario to share the spatial information for better performance [28].

As stated above, most of the approaches are based on single embryological component detection. Very few researchers focused on multi-class embryo component detection which is essentially required for collective embryological analysis with their morphological properties. The image analysis lacks research studies due to unavailability of the scientifically labeled data. Kheradmand et al. detected the three components of embryo ZP, TE, and ICM using the neural network approach. In detail, they used discrete cosine transform for the segmentation process, and a two-layered neural network is used to predict the component properties [29]. Saeedi et al. all provided the publicly available human embryo dataset with multi-class labeling by expert embryologists. They also provided the baseline methods for multi-class segmentation of these components by using a novel method of segmentation that uses texture properties along with the physical properties of the blastocyst. Finally, the segmentation refinement is achieved by the level-set method [30]. An effective deep-learning-based segmentation method is proposed by Rad et al. for multi-class blastocyst component segmentation. In detail, they used a semantic segmentation approach in which a Cascaded Atrous Pyramid Pooling (CAPP) is used for multi-scale contextual information and dense progressive sub-pixel upsampling to reproduce the resolution of the features [31]. Further artificial intelligence applications related to embryology and embryo viability prediction can be found in [32].

## 3. Proposed Methodology

**Figure 2:**
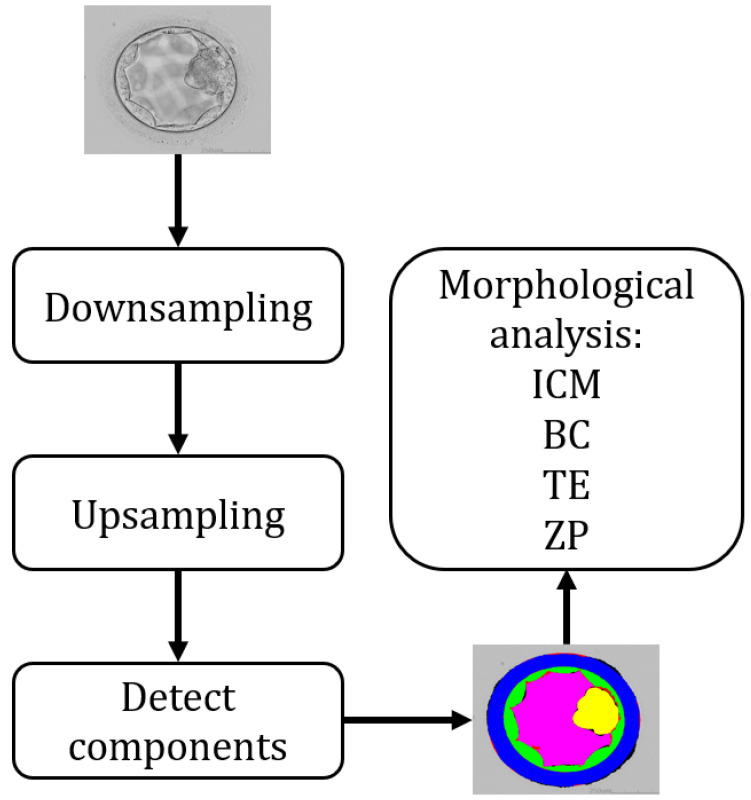
Overall summary of the proposed method.

Most of the already developed approaches deal with a single component or they are unable to provide effective segmentation performance for effective morphological analysis of the embryo. The accurate segmentation of ICM, BC, TE, and ZP provides an opportunity to analyze the morphometric properties of these components to choose the most viable embryo to improve the chances of pregnancy through IVF. To deal with these issues, this study proposes an effective deep-learning-based embryo component segmentation network (ECS-Net) that is capable to provide accurate detection of these components for embryological analysis. ECS-Net is a semantic segmentation network that provides a pixel-wise classification of these embryological components in a multi-class scenario. Figure 2 explains the overall summary of the proposed method. The ECS-Net takes the original image and applies the downsampling operation to learn the meaningful features of the classes, and then the upsampling operation to create back the original sized image. The output of the ECS-Net is four individual masks representing the detected mask of each ICM, BC, TE, and ZP. These masks can be used to analyze the morphological properties and to judge the viability of the embryo.

### 3.1. Overview of Proposed Architecture

**Figure 3:**
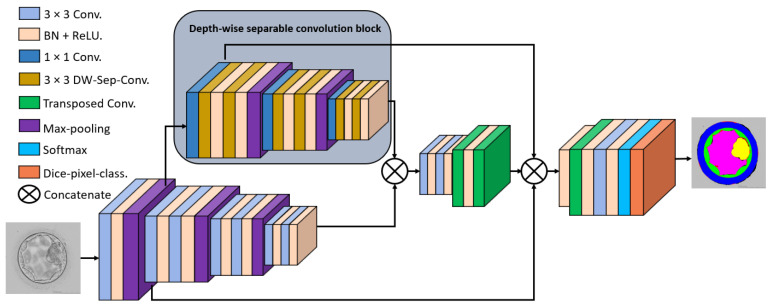
Architecture of the proposed ECS-Net.

The proposed ECS-Net is specifically designed to perform multi-class semantic segmentation for collective embryological analysis based on ICM, BC, TE, and ZP morphological properties. The ECS-Net utilizes two different feature streams and combine them using dense connectivity by depth-wise concatenation to create powerful features. As shown in Figure 3. ECS-Net uses the base convolutional stream (the lower stream), and the stream produced by a depth-wise separable convolutional block (DWSC block) (the upper stream). There are five design principles that are kept in mind to develop ECS-Net architecture. First, the deep learning architectures are prone to overfitting which deteriorate the overall performance, ECS-Net uses dense connectivity [33] that helps in reducing the overfitting. Second, the DWSC block output the upper stream that contains the low-level features that are not downsampled much, and these features compensate the spatial losses in the lower stream. Third, the upper stream is just based on depth-wise separable convolutions that help the network to reduce the number of parameters in the encoder. Fourth, unlike the conventional semantic segmentation architectures [34,35], ECS-Net does not use the same number of convolutional layers in the encoder, and the decoder follows the same design. ECS-Net is using a few layers for the upsampling of images to reduce the number of the parameters at the decoder end. Fifth, the continuous convolution causes spatial information loss, and leading to embryo components with close pixel value, therefore the two dense skip connections from the upper and lower streams help in improving the edge information for better segmentation performance. Table 1 presents the architectural differences between proposed ECS-Net, SegNet [34], and U-Net [35]. Our proposed ECS-Net has the following features:

1. Fewer convolutional layers used in combination with depth-wise separable convolutions in a two-stream custom design for accurate embryo component detection.
2. ECS-Net is empowered with dense connectivity that lets the network perform better with a shallow architecture without using any preprocessing schemes for image enhancement, where just minor morphological operations are used to clean the boundaries of the detected areas.
3. The proposed ECS-Net is providing noticeably good segmentation performance with only 2.84 million trainable parameters.

### 3.2. Working Principle of Proposed ECS-Net

**Figure 4:**
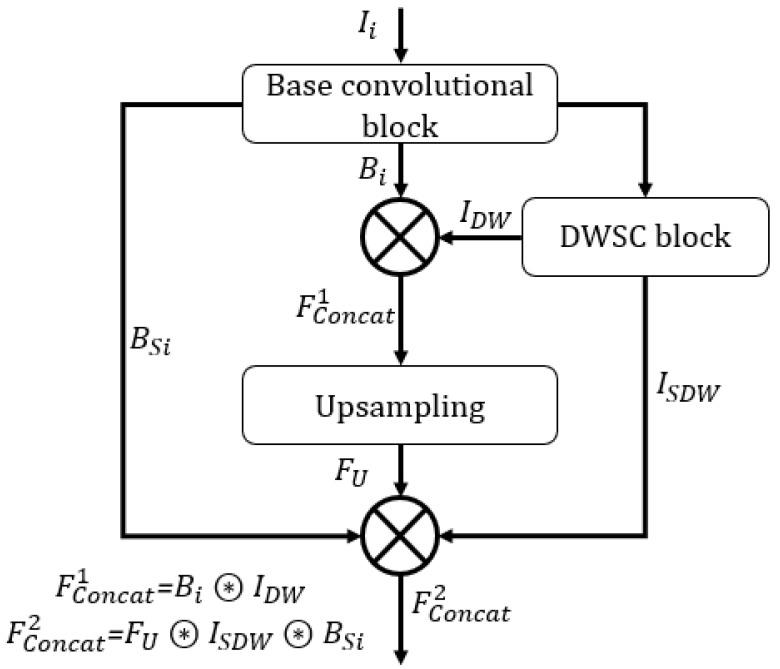
Connectivity of proposed ECS-Net.

The proposed ECS-Net is a fully convolutional network that does not use fully connected layers. The ECS-Net consists of three main blocks a main convolutional block (represented by a lower stream in Figure 3), a DWSC block (represented by an upper stream in Figure 3, and the upsampling block (represented by the right side of Figure 3). Figure 4 presents the connectivity schematic used in ECS-Net. The base convolutional block takes the feature $I_{i}$ as input from the input convolutional block and provides the $B_{i}$, and the DWSC block outputs $I_{D_{W}}$. The two features $B_{i}$ and $I_{D_{W}}$ are then depth-wise concatenated to empower the base convolutional block feature by adding low-level spatial information which results in the $F_{concat}^{1}$ feature given by Equation (1).

(1) $$F_{concat}^{1}=B_{i}\;⍟\;I_{DW},$$

Here, symbol ⍟ represents the depth-wises feature concatenation. The $F_{concat}^{1}$ feature is the resultant feature after the concatenation of the upper and lower streams. The feature $F_{concat}^{1}$ is provided to the upsampling block, where the feature is upsampled back to the original size.

Inside the upsampling block, the upsampled feature $F_{U}$ from upsampling block is further enhanced by adding spatially rich features $I_{SDW}$, and $B_{Si}$ from the DWSC block, and base convolutional block, respectively. Hence, the aggregated final feature $F_{concat}^{2}$ is produced by depth-wise concatenation of $I_{SDW}$, $B_{Si}$, and The $F_{U}$ give by Equation (2)

(2) $$F_{concat}^{2}=F_{U}\;⍟\;I_{SDW}\;⍟\;B_{Si},$$

The $F_{concat}^{2}$ feature is the resultant feature that is provided to the pixel classification block to label the pixel to each embryo component class. The pixel classification block is explained in Section 3.3.

### 3.3. ECS-Net Pixel Classification Block

The upsampling block starts right after the first concatenation in the network, and it uses a few layers to upsample the image to its original size. There is a total of three transposed convolutions in the network that revert the operation of pooling. It can be noticed from Figure 1 that there is a big difference between the number of pixels representing different classes. For example, the number of pixels in ZP class is much more than in the ICM. This difference creates a class imbalance which pushes the network to converge for the class that is overrepresented in the image. As the background pixel is with the largest contribution, this class imbalance will cause deterioration of detection performance. The Generalized Dice Loss (*GDL*) [36] is an effective solution to deal with the class imbalance and provide accurate segmentation performance. *GDL* limits the contribution of each class in the loss by weighting it according to the inverse size of the region. The *GDL* is given by Equation (3)

(3) $$L_{GDL}=1-2\;\frac{\sum_{i}^{K}P_{ci}G_{ci}+\epsilon }{\sum_{i}^{K}P_{ci}^{2}+\sum_{i}^{K}P_{ci}^{2}+\epsilon }$$

where *K* is the set of all pixel locations available in the image, *i* is the candidate pixel, and *P* and *G* define the prediction by the classifier and ground truth, respectively, where $P_{ci}$ is the predicted probability of pixel i which is belonging to candidate class *c*, and $G_{ci}$ is the actual class label in the ground truth.

At the output, the ECS-Net provides five masks, which represent ICM, BC, TE, ZP, and background class. Each individual mask represents that specific class by "1" and the others by zero. The ECS-Net is trained in a multi-class scenario, and it provides all the five predicted masks in one go.

## 4. Experimental Results

This section presents the experimental results and evaluation of the proposed method. The ECS-Net experiments were conducted on a desktop computer with Intel(R) i7-7500U CPU 3.50 GHz processor, 32 GB RAM, and Nvidia Geforce GTX-1070 GPU on MATLAB 2021b. The implementation hyper-parameters are mentioned in Table 2. The ECS-Net is specifically designed for embryo component segmentation for embryological analysis. The proposed ECS-Net is trained from scratch without using any weight initialization, migration, fine-tuning or transfer learning from other networks.

### 4.1. Dataset and Augmentation

In this paper, we used the publicly available microscopic human blastocyst dataset introduced by the study [30]. The dataset contains 235 microscopic dataset captured by Olympus inverted microscope. These images are collected at Pacific Center for Reproduction Canada between the years 2012 and 2016. The expert labeling of the embryo component was provided with the dataset for supervised learning and evaluation of the algorithms. In our experiments, from the total of 235 images, we used 85% images (200 images) for training and the rest of 15% images (35 images) for testing. Moreover, the medical images are usually in low quantity, these low numbers of images are not sufficient to properly train a deep learning network. To prevent overfitting and to achieve better training of the ECS-Net we utilized image augmentation to increase the number of training images. In detail, we used flipping (horizontal, vertical), rotation, crop, and resize transformations to create 3200 images.

### 4.2. Evaluation Criteria

The proposed ECS-Net takes the original image and provides the individual masks for each ICM, BC, TE, and ZP component of the human embryo. To fairly compare and evaluate the ECS-Net with existing schemes, we used Jaccard Index (Jaccard) as an evaluation metric given by Equation (4). The Jaccard is similar to the intersection of union (IOU) which is a versatile segmentation evaluation metric. The computation of Jaccard is based on the expert label comparison with the predicted label mask for each ICM, BC, TE, and ZP class by counting the true positive (*TP*), false negative (*FN*), and false-positive (*FP*) pixels, which are described as follows.

1. True Positive (*TP*): is the pixel that belongs to the embryo component in both the predicted mask and expert label mask;
2. False Negative (*FN*): is the pixel that is incorrectly predicted as a background pixel where it is actually an embryo component pixel in the expert label mask;
3. False Positive (*FP*): is the pixel which is incorrectly predicted as embryo component pixel, where actually it belongs to a background pixel in expert label mask.

(4) $$Jaccard=\frac{\#TP}{\#TP+\#FN+\#FP}$$

### 4.3. Comparison with State-of-the-Art

**Figure 5:**
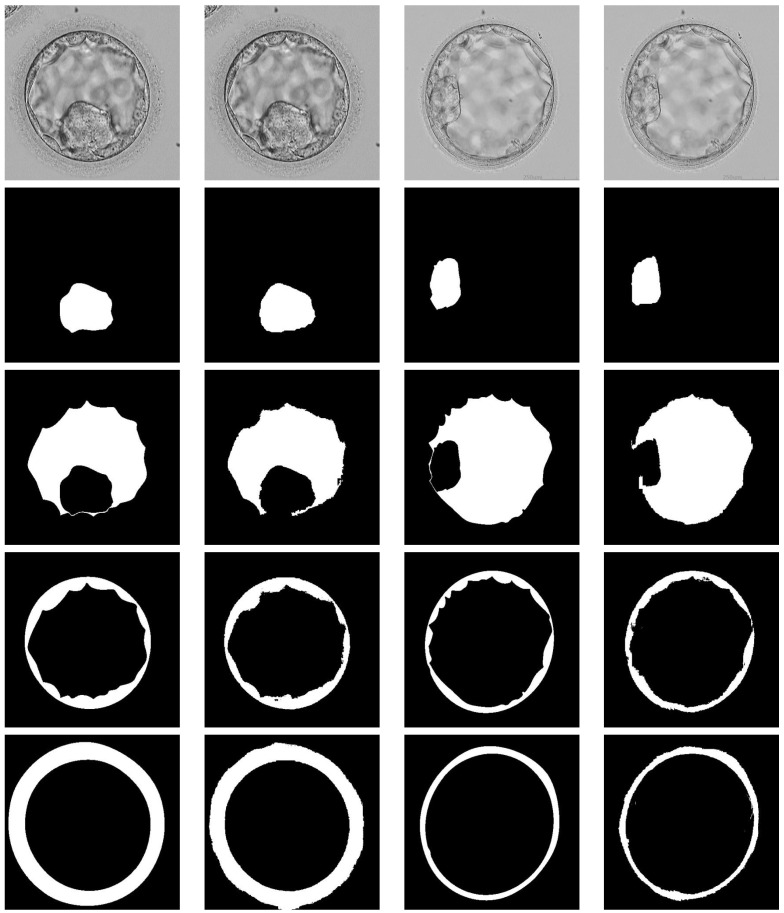
Visual results for separate embryo components segmentation. From left-to-right column: Example-1 images with ground truth, Example-1 Predicted results by ECS-Net, Example-2 images with ground truth, and Example-2 Predicted results by ECS-Net.

**Figure 6:**
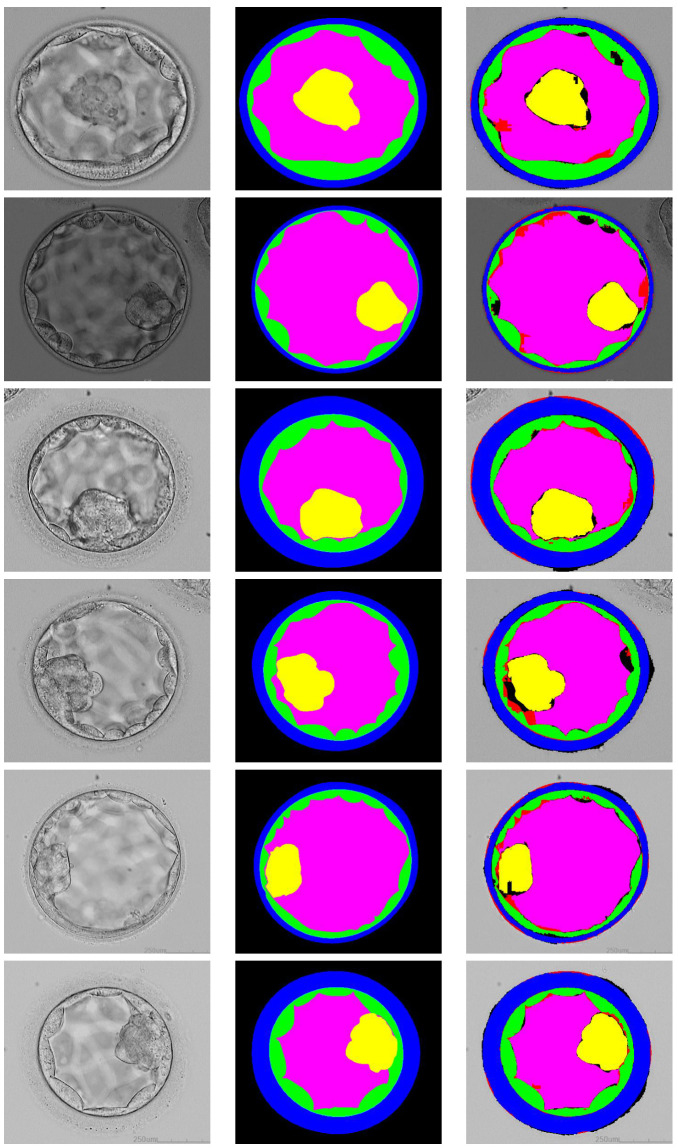
Visual results of embryo components combined segmentation using ECS-Net. From left-to-right: input images, expert label mask, segmentation by proposed ECS-Net.

This section provides the visual and numerical results of the proposed ECS-Net. The proposed network generates four separate predicted masks for each ICM, BC, TE, and ZP class. Figure 5 represents the segmentation results of each embryo component with an expert annotation label. Where Figure 6 shows the combined results for the overall embryo with an expert annotation mask. Table 3 shows the numerical results based on Jaccard provided in Equation (4), and it also provides the comparison of ECS-Net with existing approaches for embryo multiclass segmentation. it can be noticed from the Table 3 that, our proposed ECS-Net is providing good segmentation performance with least number of trainable parameters.

### 4.4. Limitation of the Proposed Method

The proposed ECS-Net is a learning-based method which learns from the annotated medical data provided by the expert embryologist. The learning accuracy of the network depends on the amount of training data. In case of embryo images, it is very difficult to arrange the large number of annotated data. Therefore, data augmentation is essentially required to artificially increase the amount of data for better training of ECS-Net.

## 5. Embryological Analysis

The proposed ECS-Net provides the accurate pixel-wise segmentation of ICM, BC, TE, and ZP. All these component’s morphologies are important to test the viability of the embryo to increase the chances of pregnancy using in vitro fertilization. The morphometric properties of each component play a vital role [11,12,13,14,15], and ECS-Net provides valuable insight into the morphometric properties of these components. The proposed masks can provide the area, size, and location of these components. The blastocyst stage creation shows that the embryo is ready to transfer, where the ZP thickness reduction can show this process, and it can be monitored using the proposed ECS predicted mask. The ECS-Net is the supervised method that learns the valuable features from the training data considering the expert labels which shows that it learns the knowledge of the expert embryologist. Therefore, such types of algorithms can benefit to check the viability of the embryo remotely.

## 6. Conclusions

In vitro fertilization is the process that is dealing with infertility effectively. The embryo viability check is essential to increase the chances of pregnancy. Single embryo transfer is considered more useful to reduce the chance of multiple pregnancies. The embryo viability monitoring is very important to increase the chance of pregnancy using single embryo transfer. The embryologist checks the morphological properties of embryo components and collectively decides the viability of the embryo. The proposed ECS-Net is a fully convolutional network that performs the multiclass semantic segmentation of these components. The ECS-Net is based on two separate streams of base convolutional block and depth-wise separable convolutional block, and these streams form valuable rich features. The ECS-Net is providing considerably good segmentation performance without preprocessing. The ECS-Net provides the accurately predicted mask for ICM, BC, TE, and ZP for embryological analysis. ECS-Net is evaluated on a publicly available human embryo dataset and provides superior segmentation performance compared to the existing state-of-the-art methods in terms of accuracy and computational efficiency. The proposed method can be used as a second opinion system to test the viability of the embryo to increase the success rate of in vitro fertilization. In the future, we will further optimize the network with enhanced segmentation performance for medical image analysis.

## Figures and Tables

**Table 1:** Comparison between proposed ECS-Net, SegNet [34], and U-Net [35].

ECS-Net (Proposed)	SegNet [34]	UNet [35]
**1.** The shallow decoder is different from encoder which overall reduced number of trainable parameters	The decoder is same as encoder (doubles the number of trainable parameters)	The decoder is same as encoder (doubles the number of trainable parameters).
**2.** Overall 10 convolutions are used	Overall 26 convolution layers	Overall 23 convolutions
**3.** Internal and external dense connectivity is used	No connectivity is used between layers	External dense connectivity is used
**4.** Three transposed convolutions are used for upsampling	Unpooling layers are used for upsampling	Four up-convolutions are used for upsampling
**5.** 2.84 million trainable parameters	29.4 million training parameters	31.03 million trainable parameters

**Table 2:** Summary of training hyper-parameters.

Training Hyper-Parameter	Value
Solver	Adam [37]
Initial-learning rate (ILR)	0.001
Normalization	Global L2
Iterations	4100
Mini-batch size	12
Image shuffling	Yes

**Table 3:** Numerical performance comparison of proposed ECS-Net with existing approaches for embryo component segmentation.

Method	ICM	BC	TE	ZP	Background	Mean Jaccard	Parameters
U-Net baseline [35]	79.03	79.41	75.06	79.32	94.04	81.37	31.03 M
TernausNet [38]	77.58	78.61	76.16	80.24	94.50	81.42	10.0 M
PSP-Net [39]	78.28	79.26	74.83	80.57	94.60	81.51	35 M
DeepLab-V3 [40]	80.60	78.35	73.98	80.84	94.49	81.65	40.0 M
Blast-Net [31]	81.07	80.79	76.52	81.15	94.74	82.85	25.0 M
ECS-Net (Proposed)	85.26	88.41	78.43	85.34	94.87	86.46	2.83 M

## Data Availability

Not applicable.
